# Electrochemical Promotion of CO_2_ Hydrogenation Using a Pt/YSZ Fuel Cell Type Reactor

**DOI:** 10.3390/nano13131930

**Published:** 2023-06-25

**Authors:** Andriana Lymperi, Christos Chatzilias, Fotios Xydas, Eftychia Martino, Georgios Kyriakou, Alexandros Katsaounis

**Affiliations:** 1 Department of Chemical Engineering, University of Patras, 26504 Patras, Greece; lymperi@chemeng.upatras.gr (A.L.); chatzilias@chemeng.upatras.gr (C.C.); up1068967@upnet.gr (F.X.); martino@chemeng.upatras.gr (E.M.); kyriakg@upatras.gr (G.K.); 2 School of Sciences and Engineering, University of Nicosia, Nicosia 2417, Cyprus

**Keywords:** EPOC, CO_2_ hydrogenation, RWGS reaction, NEMCA, SOFC type reactor

## Abstract

The hydrogenation of CO_2_ is a reaction of key technological and environmental importance, as it contributes to the sustainable production of fuels while assisting in the reduction of a major greenhouse gas. The reaction has received substantial attention over the years within the catalysis and electrocatalysis communities. In this respect, the electrochemical promotion of catalysis (EPOC) has been applied successfully to the CO_2_ hydrogenation reaction to improve the catalytic activity and selectivity of conductive films supported on solid electrolytes. However, designing an effective electrocatalytic reactor remains a challenge due to the connections required between the electrodes and the external potentiostat/galvanostat. This drawback could be alleviated if the catalytic reaction occurs in a reactor that simultaneously operates as a power generator. In this work, the Electrochemical Promotion of the CO_2_ hydrogenation reaction in a low-temperature solid oxide electrolyte fuel cell (SOFC) reactor is studied using yttria-stabilized zirconia (YSZ) and a platinum (Pt) electrode catalyst. The system has been studied in two distinct operation modes: (i) when the necessary energy for the electrochemical promotion is produced through the parallel reaction of H_2_ oxidation (galvanic operation) and (ii) when a galvanostat/potentiostat is used to impose the necessary potential (electrolytic operation). The performance of the fuel cell declines less than 15% in the presence of the reactant mixture (CO_2_ and H_2_) while producing enough current to conduct EPOC experiments. During the electrolytic operation of the electrochemical cell, the CO production rate is significantly increased by up to 50%.

## 1. Introduction

For the first time in history, humanity is confronted with a major crisis related to climate change, energy demands, and power sources. The continuous human population growth, together with the carbon-based energy economy, has led to a striking production of greenhouse gases. The dominant greenhouse gas, carbon dioxide (CO_2_), has increased by 149% since the pre-industrial period, reaching values higher than 410 parts per million in 2020, according to the World Meteorological Organization (WMO) Greenhouse Gas Bulletin [1]. The rise of carbon dioxide emissions due to anthropogenic activity has led policymakers, the scientific community, and global industry to focus on effective methods of controlling and managing processes associated with CO_2_ emissions. 

The direction of geologic sequestration of CO_2_ involves risks, such as inadequate underground volumes for the necessary CO_2_ quantities to reach net negative emissions [2]. In addition, the risk of leakage, the induced seismicity, and the cost of CO_2_ capture from the stationary power plants [3,4] are additional factors that lead the community to study alternative routes of CO_2_ utilization, mainly to the transformation of CO_2_ into value-added chemicals and high energy fuels, mainly through the reaction of CO_2_ with hydrogen [5,6,7,8].

Therefore, the production of useful fuels from the CO_2_ hydrogenation reaction (such as methane, syngas, methanol, ethanol, formic acid, etc. [5,9,10]), has evolved into an important research area, including the challenges associated with it like physical (sintering, fouling, and attrition) and chemical deactivation (poisoning or vapor-solid reaction) of the catalyst [10,11], the origin of the necessary for the reaction hydrogen [10], as well as the reactor operating conditions [7].

The Reverse Water-Gas Shift reaction (RWGS) is an important reaction that may occur over a hydrogenation catalyst when co-feeding of CO_2_ and H_2_:

(1) $$CO_{2}+H_{2}\to CO+H_{2}O\Delta H_{r,298K}^{o}=41.5\frac{kJ}{mol}$$

The above reaction, leading to syngas production, is mildly endothermic and favored at high temperatures. The inability to utilize active RWGS catalysts at low temperatures due to thermodynamic limitations (CO production is predicted at temperatures higher than 700 °C) [12] poses a problem. Two are the main popular mechanisms suggested in the literature for the RWGS reaction, which comprehensively depend on whether the dissociated H_2_ species are involved in the formation of the carbon-containing intermediates [13]. The first one (surface redox mechanism) involves the oxidation of the catalyst through the dissociative adsorption of the CO_2_ to carbonyl (CO_ads_) and O_ads_, followed by the desorption of CO and catalyst reduction by dissociatively adsorbed hydrogen (H_ads_) to form H_2_O. The second mechanism (an associative mechanism) proposes that the adsorbed on the catalytic surface CO_2_ reacts directly with dissociatively adsorbed hydrogen (H_ads_) to form intermediate species, such as formate (HCOO), carboxyl (COOH), and bicarbonate (HCO^3−^), which is then decomposed to CO and H_2_O [14]. 

Further to the RWGS reaction, CO_2_ hydrogenation is a synthesis reaction leading to the formation of a range of hydrocarbons or/and alcohols according to Equation (2):

(2) $$xCO_{2}+\left(2x-z+y/2\right)H_{2}\to C_{x}H_{y}O_{z}+\left(2x-z\right)H_{2}O$$

Reaction (2) is usually exothermic, favored at lower temperatures, and the derived products depend on the reaction conditions (temperature, pressure, etc.) as well as the type of the used catalyst.

CO_2_ reduction to valuable fuels via heterogeneous catalytic processes has been thoroughly studied using various active metals and supports. Noble metals, including Ru, Rh, Pt, and Pd [5,15,16], as well as inexpensive metals such as Ni, Cu, and Co [17,18,19,20], deposited on various supported materials, have been thoroughly discussed in the literature for their catalytic and electrocatalytic performance in terms of activity and selectivity in a plethora of reactors such as fixed-bed, single-chamber, monolithic-type and SOFC type reactors [19,21,22,23,24,25,26,27,28]. Among these, Pt catalysts perform a strong activity in H_2_ adsorption, high corrosion resistance, low coke deposition, as well as high selectivity to the RWGS reaction [29]. In this respect, the electrochemical promotion of catalysis (EPOC) can be utilized to increase the overall CO_2_ conversion to valuable products. EPOC has been used extensively in the past for the modification of metal catalyst work function and thus resulting in notable activity and selectivity change over various catalytic reactions [30,31,32]. The utilization of electronegative or electropositive promoters/supports in conjunction with current or interfacial potential application leads to the desired catalytic activity and selectivity modification [33].

EPOC has been applied in numerous studies for the CO_2_ hydrogenation reaction [20,21,23,24,25,34,35,36,37] both in laboratory-scale single chambers as well as in semi-pilot reactors. In both cases, there are design drawbacks mainly associated with the reactor/cell configuration and the wiring of the electrodes with the external energy source (i.e., potentiostat/galvanostat). Thus, the application of EPOC on an industrial scale is limited. A potential solution to the aforementioned problems will be the simultaneous production of the necessary power for the electrochemical promotion of a desired reaction in the reactor itself. 

A fuel cell type configuration, where the chemical energy of a fuel is directly converted into electricity, could play such a role. To this day, a plethora of such electrochemical devices have been reported, the most popular among them being the polymer electrolyte membrane fuel cells (PEMFCs), Alkaline fuel cells (AFCs), phosphoric acid fuel cells (PAFCs), and molten carbonate fuel cells (MCFCs) [38,39]. The first four types are properly operating at temperatures lower than 250 °C, where the investigated reaction (i.e., RWGS) is not feasible to occur due to thermodynamic and kinetic restrictions. The MCFC type uses carbonate ions (CO_3_^2−^) as a conductor, which can conflict with the reactant mixture (CO_2_ and H_2_). Solid oxide fuel cells (SOFC) are one of the most attractive cell types due to their low-cost solid materials, long-term operation, and economical maintenance [40,41], as well as due to the moderate operation temperature range, which is suitable for the RWGS reaction. Moreover, green hydrogen utilization via fuel cells (i.e., hydrogen produced by clean energy derived from renewable energy sources [42]) meets the zero-emission of many global and national associations targets set for 2050. Although the use of a solid oxide fuel cell reactor for the investigation of EPOC has a lot of benefits, only a limited number of studies have been reported [21,22,27,28]. Chatzilias et al. reported significant electrochemical promotion of the CO_2_ hydrogenation reaction to CH_4_ and CO using a low-temperature tubular SOFC reactor based on Ru film as a working electrode [21]. Bebelis et al. [27] reported the use of tubular SOFC consisting of Rh-working electrodes to electrochemically promote the methanation and RWGS reaction. In addition, the study of the electrochemical promotion of the RWGS over Pt on K^+^-β″-Al_2_O_3_ at a bench scale reactor was performed by Ruiz et al. [28]. Additionally, the electropromotion of the RWGS reaction using a tubular SOFC reactor based on Pt/YSZ/Pt was studied in a temperature range of 650–800 °C by Pekridis et al. [22].

**Scheme 1:**
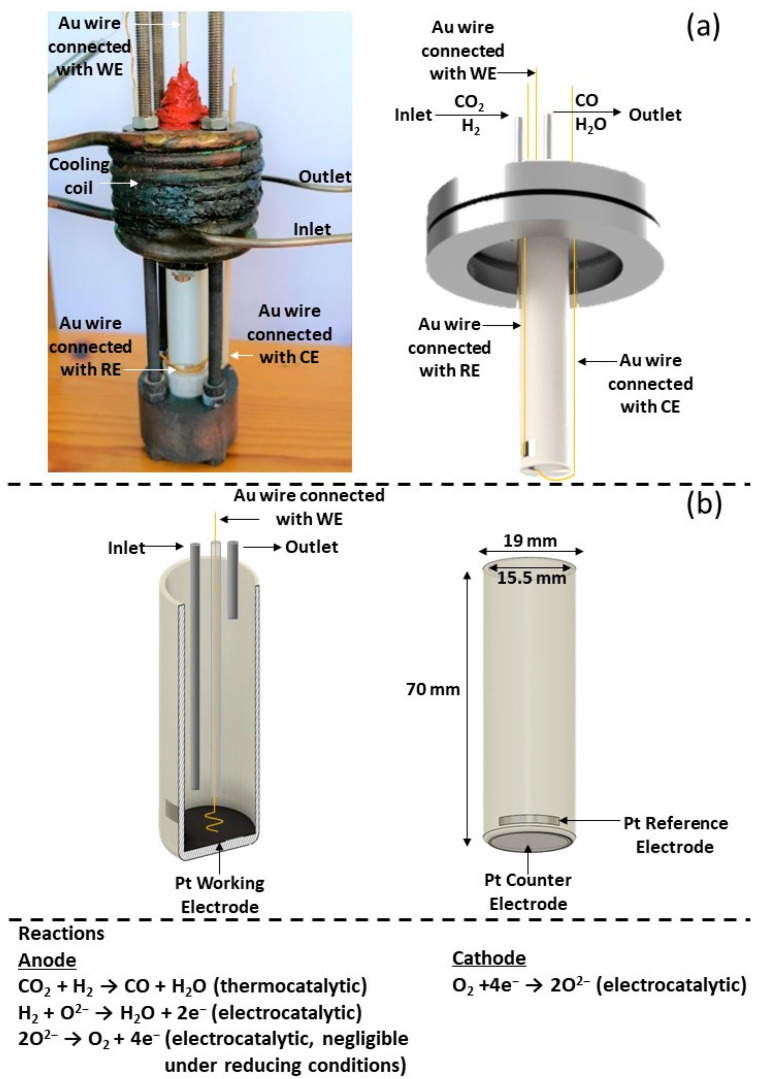
(**a**) Real and schematic representation of the tubular SOFC reactor. (**b**) Cross section of the YSZ tube with the Pt working electrode (anode). External view of the YSZ tube with the Pt reference and Pt counter (cathode) electrodes and the Au wire connections. Corresponding reactions occur to each electrode.

Herein, we introduce a robust low-temperature tubular SOFC-type reactor where the principles of EPOC are being applied (Scheme 1). The CO_2_ hydrogenation reaction, occurring on the anodic catalytic surface area, is electrochemically promoted either using power generated by the fuel cell (galvanic mode) or using external power provided by a potentiostat/galvanostat (electrolytic mode). It is worth noting that although the most efficient operating temperature of SOFCs often ranges between 800–1000 °C [43], the SOFC temperature operation of the present study is remarkably limited to lower temperatures. This is directly connected with the necessity for the electrochemical promotion power values, which are feasible even at low temperatures (<500 °C). To the best of our knowledge, there are no relevant studies in the literature which utilize fuel-cell-type reactors with Pt as working/catalyst and counter electrodes for the electrochemical promotion of the RWGS reaction at low-temperature regimes.

## 2. Experimental 

### 2.1. Pt/YSZ/Pt Cell Preparation

The solid electrolyte component (Sheme 1), provided by Everspring Ceramic (Shanghai, China), was of a tubular geometry consisting of an 8% mol Y_2_O_3_ stabilized ZrO_2_ (YSZ) disk. The inner and outer diameters were 15.5 mm and 19 mm, respectively, with a height of 70 mm and a bottom thickness of 1.4 mm (Scheme 1b). The Pt counter electrode (CE) (outer bottom) and the Pt reference electrode (RE) (outer sidewall close to the bottom) were deposited via DC magnetron sputtering. The tubular YSZ reactor was initially placed in a sputtering chamber filled with pure Argon with a pressure of 0.5 Pa. Then, metal Pt (purity 99.999%) was deposited onto the substrate at 30 °C with an applied direct current (DC) of 0.5 A and discharge of 650 V. Τhe Pt catalyst film, which simultaneously was used as working electrode (WE), was prepared on the inner bottom of the YSZ tubular reactor in a two-step process: first, an H_2_PtCl∙6H_2_O (Honeywell, Charlotte, NC, USA) 100 mM solution in isopropanol was deposited dropwise at 35 °C followed by calcination at 500 °C in air for 2 h; and second, via a reduction pretreatment in 15% H_2_/He at 400 °C for 3 h. The resulting mass of the catalytic film was 2.9 mg.

### 2.2. Catalyst Characterization

The catalyst characterization was carried out by XRD using a Siemens D500 diffractometer (Frankfurt, Germany) and Cu Ka radiation. The range of scanning angles was 2θ = 20–80°, and the wavelength of the incident radiation was λ_CuKa_ = 1.542 Å. Identification of major peaks was obtained after comparison with library values (JPCDS). 

X-ray photoelectron spectroscopy (XPS) measurements were carried out in a system described in detail elsewhere [44]. Briefly, the system consists of non-monochromatic AlKa radiation (1486.6 eV) and a Leybold LH EA11 energy analyzer (Leybold, Germany), which was operated at a constant pass energy (100 eV) and pressure (2 × 10^−9^ mbar). The analyzed sample area was 2 × 5 mm^2^ rectangle. All spectra were corrected for charge transfer using the C1s peak at 284.8 eV for adventitious carbon. 

### 2.3. Reactor Operation

A metallic stainless-steel head that includes a stainless-steel water-cooling coil, built-in streams for the inlet and outlet mixture, three current collectors for the electrodes, and a glass socket for a K-type thermocouple, was connected with the YSZ tubular reactor using a Viton O-ring to secure the connection (Scheme 1a). The water-cooling coil of the metallic head was used to maintain the low temperature of the O-ring in order to prevent any gas leak. In addition to the K-type thermocouple, which was placed inside the reactor, close to the catalytic film (almost attached), a second thermocouple positioned in the exterior bottom of the reactor was used to confirm the reaction’s temperature as well as to measure any temperature fluctuations. The reactor was placed in a quartz furnace. The bottom of the YSZ tubular reactor was in the middle of the furnace to achieve a uniform temperature on the catalytic surface, while the metallic head was outside of the furnace.

The gas feed composition and total gas flow rate were electronically controlled using a set of flow meters provided by Brooks Instrument (Hatfield, PA, USA). High-purity compressed gases provided by Linde Hellas were used to compose the anode reaction mixture. Specifically, certified 5% CO_2_ in He, 15% H_2_ in He as well as pure He 99.999% gas were combined in order to achieve various P_CO_2__:P_H_2__ ratios (1:7 and 4:1) and the desired flow rate levels (100–400 cm^3^/min). The cathode compartment was exposed to the room atmospheric air at ambient pressure, and thus the required oxygen for both the electrochemical promotion and fuel cell operation is provided without any additional mass flowmeter. All experiments were performed under atmospheric pressure and temperatures ranging between 260–460 °C. 

For the quantitative and qualitative analysis of the outlet mixture of the anodic compartment, an online gas chromatography (Shimadzu GC-2010 Plus, Kyoto, Japan), equipped with two FS, ValcoPLOT, Molesieve, and Alumina columns) in conjunction with an infrared gas analyzer (Fuji Electric, ZRE-3, Shinagama City, Tokyo, Japan) with four-channel detection capability (CO_2_, CO, CH_4_, O_2_) were used. An AMEL 7050 potentiostat/galvanostat, connected to the three electrodes through Au wires, as shown in Scheme 1, was used to apply constant currents and potentials. The electrochemical investigation of the CO_2_ hydrogenation reaction was carried out under two modes: electrolytic mode of the reactor, i.e., the energy required for polarization was provided by an external source (potentiostat/galvanostat), and galvanic mode, i.e., the required energy was obtained directly from the parallel operation of the fuel cell.

For the presentation of the results and, in particular of the values of the potentials, the following conventions were used; symbol U_fc_ was used for the potential difference between cathode and anode (U_cathode_–U_anode_), according to the IUPAC standards, and symbol U was used for the potential difference between anode and cathode (U_anode_–U_cathode_). The potential difference U can be used to better understand the oxygen ion direction (i.e., from or to the Pt catalyst-working electrode) as well as to easily compare the current results with previous EPOC studies. Under this convention (electropromoted conditions), the fuel cell open circuit potential corresponds to ~−1.2 V, and the application of any additional potential is either added or subtracted from this value.

## 3. Results and Discussion

### 3.1. Physicochemical Characterization

**Figure 1:**
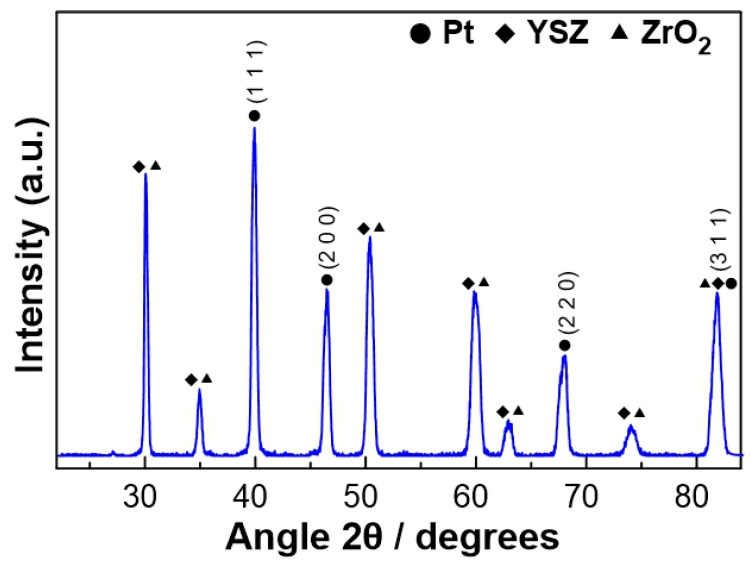
XRD diagram of the Pt catalytic film deposited on YSZ after reduction treatment.

The characterization of the fresh (prior to experiments) Pt catalyst, after the reduction step, was carried out by XRD in order to obtain information about the crystallinity of the platinum catalytic film. As shown in Figure 1, well-defined reflections were revealed at angles 2θ = 39.9, 46.5, 67.8, and 81.5°, which correspond to the metallic Pt phase. The remaining peaks shown in Figure 1 were attributed to the YSZ solid electrolyte.

The average size of the Pt crystallites was estimated using Scherrer’s [45] equation:

(3) $$L=\frac{k\cdot \lambda }{\beta \cdot cos(\theta )}$$

where L is the average size of the ordered (crystalline) domains, which can be less than or equal to the particle size (nm), λ is the wavelength of the incident beam (nm), β is the width of the diffraction peak at half its height (rad), θ is the angle between the incident beam and the reflecting plane (Bragg angle), and K is a dimensionless constant with a value of 0.9. Based on the peaks observed at the angles 2θ = 39.9, 46.5, 67.8, and 81.5°, the size of the Pt crystallites was estimated to equal 14.3 ± 1.8 nm.

**Figure 2:**
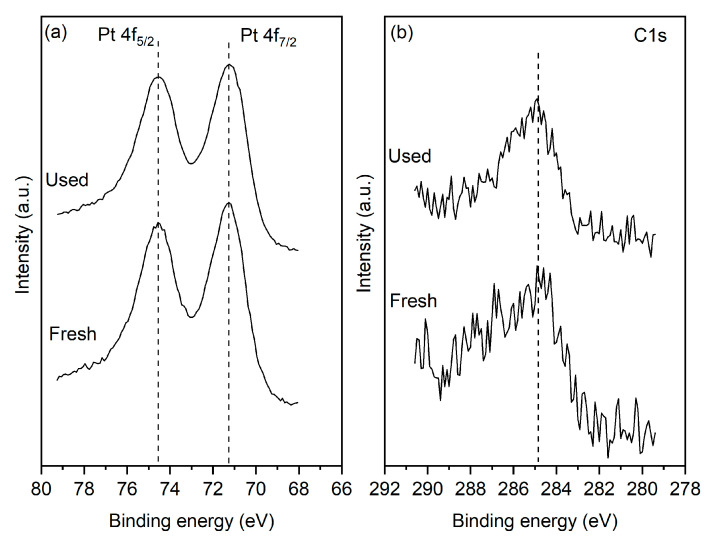
(**a**) Pt 4f and (**b**) C1s spectra of the fresh and used sample.

XPS measurements were conducted on the fresh reduced sample and after 25 h of operation under oxidizing (i.e., P_CO_2__:P_H_2__ = 4:1) conditions. Figure 2a shows that for both the fresh and used samples, the Pt 4f_7/2_ peak is centered at 71.2 eV, which is characteristic of metallic Pt. The results indicate that after the reaction, the catalyst film retains its metallic state. More importantly, the reaction does not lead to any increase in the carbon content of the surface. The C1s signal shown in Figure 2b indicates the presence of adventitious surface carbon on both the fresh and used samples. Crucially the carbon content of the surface does not increase for the post-reaction sample. It is worth noting that the XPS measurement shows only trace quantities of Cl_2p_, suggesting that the sample treatment has removed most of the chlorine present in the precursors which were used to prepare the films.

### 3.2. Electrocatalytic Results

**Figure 3:**
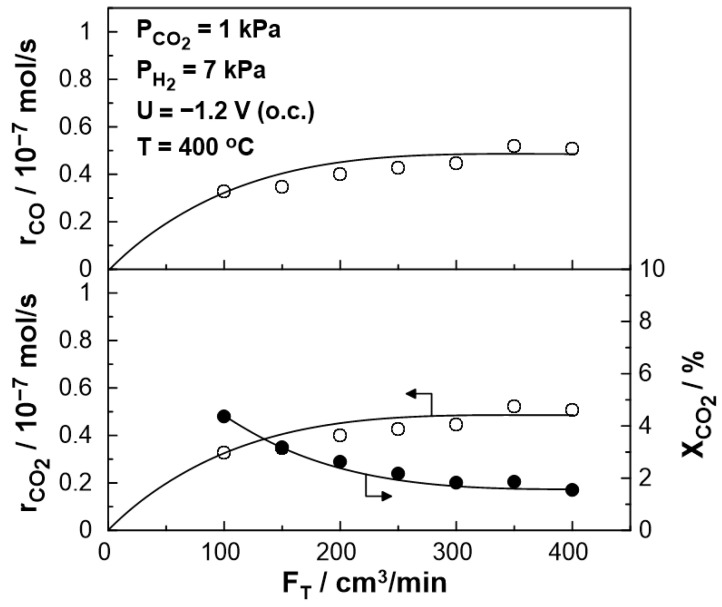
Effect of the total gas flow rate, F_T_, on CO formation catalytic rate (top), on CO_2_ reduction rate (bottom left), and CO_2_ conversion (bottom right), under open circuit conditions (U_fc_ = 1 V). P_H_2__ = 7 kPa, P_CO_2__ = 1 kPa, T = 400 °C.

The steady-state effect of the total gas mixture flow rate on the catalytic rate and CO_2_ conversion at 400 °C is shown in Figure 3. The catalytic rate of CO production initially increases and, for a wide range between 200 and 400 cm^3^/min, reaches a plateau. Thus, mass transfer limitations are negligible for gas flow rates higher than 200 cm^3^/min. This value was selected for the rest of the experiments. 

**Figure 4:**
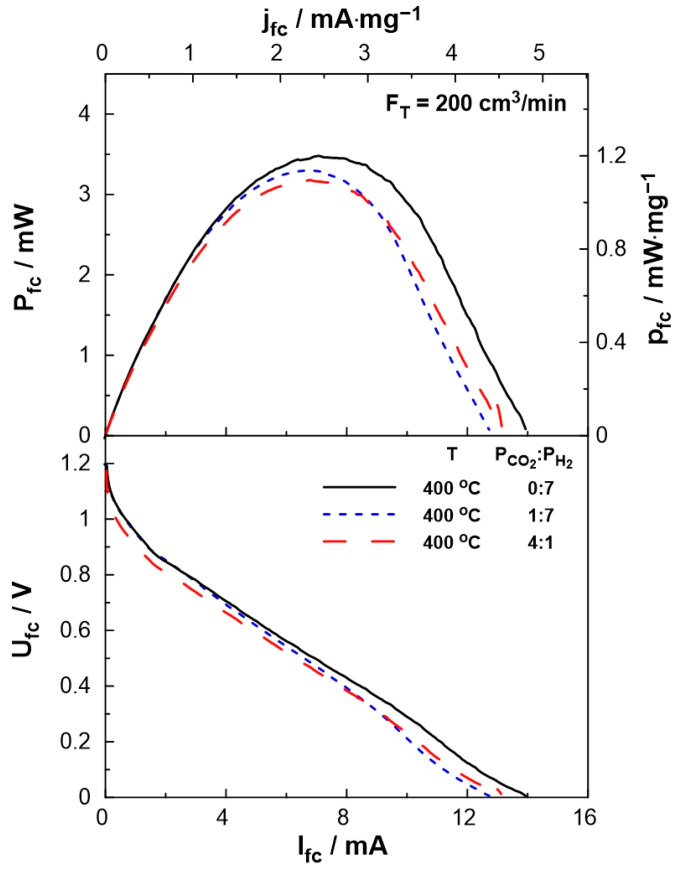
SOFC current-power, I_fc_–P_fc_, (**top**) and current-potential, I_fc_–U_fc_, (**bottom**) curves for different P_CO_2__:P_H_2__ (=0:7, 1:7, 4:1) ratios. Solid curves correspond to a single operation of the cell, while dashed curves correspond to a dual operation (see text). F_T_ = 200 cm^3^/min, T = 400 °C.

The performance of the SOFC-type reactor was evaluated both under fuel cell operation, i.e., using H_2_ as the unique reactant, and dual operation, i.e., during co-feeding of H_2_ and CO_2_ at the anode of the reactor. Figure 4 shows the effect of current on the cell potential and power output. The experiments were carried out at a constant temperature of T = 400 °C and various reactant ratios (P_CO_2__:P_H_2__ = 0:7, 1:7, 4:1). The maximum current (I_fc_ = 14 mA), as well as the maximum power output (P_fc_ = 3.5 mW), were observed under fuel cell operation. Under dual operation, the presence of CO_2_ on the anode leads to a small decrease in the performance of the fuel cell, which is less than 15%, even at high partial pressures of CO_2_ (4 kPa). It is worth mentioning that although the observed current values are lower than those produced in conventional fuel cells (as expected due to the low operation temperature), they are within the same range as those applied in EPOC experiments [46,47] and can be utilized for the electrochemical promotion of the catalytic reaction. 

**Figure 5:**
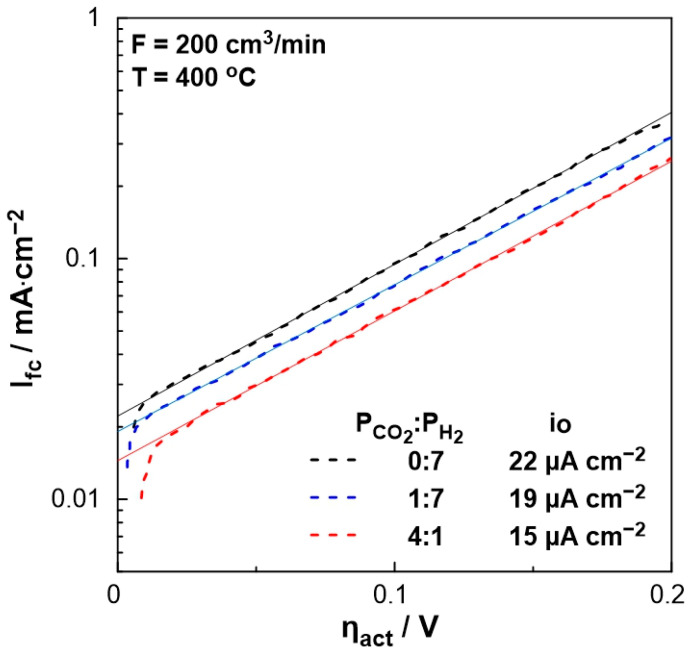
The logarithm of the current density, I_fc_, as a function of the overpotential, η_act_, for different P_CO_2__:P_H_2__ (=0:7, 1:7, 4:1) ratios. F_T_ = 200 cm^3^/min, T = 400 °C.

In order to evaluate the polarizability of the catalyst (working electrode), which is associated with its capability for electrochemical promotion, the exchange current density, i_o_, and charge transfer coefficient, α, were estimated under different P_CO_2__:P_H_2__ ratio feeding conditions. Figure 5 shows the current density produced by the fuel cell as a function of the activation overpotential (Tafel diagram—dashed lines) for the region of activation polarization (U_fc_ > 1 V in Figure 4). The intercept of the tangent lines on the aforementioned curves (solid lines) corresponds to the exchange current density, i_o_, which varies with the feed gas mixture ratio. More specifically, in the absence of CO_2_, the exchange current density is higher than that obtained under strongly reducing conditions (P_CO_2__:P_H_2__ = 1:7), whereas it is the lowest under oxidizing conditions. Thus, the stronger the reducing conditions, the more polarizable the metal-solid electrolyte interface. Moreover, the charge transfer coefficient, α, which is equal to the slope of the solid lines in Figure 5, remains constant (α = 0.4), indicating that the mechanism of the electrochemical reactions does not change by the ratio of reactants.

**Figure 6:**
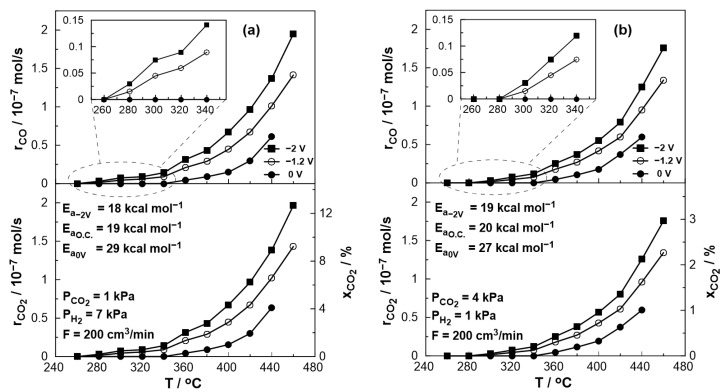
Effect of temperature on the steady-state catalytic rates for CO production (**top**), CO_2_ reduction (**bottom**, left axis), and conversion (bottom, right axis) under open circuit (U= −1 V), short circuit (U = 0 V) and negative polarization (U = −2 V) conditions. (**a**) P_H_2__ = 7 kPa and P_CO_2__ = 1 kPa. (**b**) P_H_2__ = 1 kPa and P_CO_2__ = 4 kPa. F_T_ = 200 cm^3^/min.

Figure 6 depicts the thermocatalytic and electropromoted rates of CO production and CO_2_ consumption and conversion at a temperature range between 260 and 460 °C under both reducing (P_CO_2__:P_H_2__ = 1:7) and oxidizing conditions (P_CO_2__:P_H_2__ = 4:1). One can observe that the catalytic rate of CO production increases with temperature, which is consistent with the thermodynamic predictions since the RWGS reaction is slightly endothermic, whereas, for temperatures above 380 °C, this increase is almost monotonous in both oxidizing and reducing conditions. It is also observed that under reducing conditions, CO production begins at a lower temperature, and the catalytic rate is slightly higher compared to the oxidizing conditions, which is in agreement with thermodynamic analysis [48]. The thermocatalytic rate was monitored under open circuit (o.c.) conditions, in which no electrochemical reactions occur, the value of current is zero, and the open circuit potential was close to the theoretical value of a hydrogen fuel cell predicted by Nernst’s Equation (U~−1.2 V). On the other hand, by short-circuiting the anodic and cathodic electrode using an external cable (U = 0 V), the electrons produced by the oxidation of H_2_ flow through the circuit, creating a condition similar to a positive polarization via external devices (e.g., potentiostat) in EPOC studies [21,23,46,47]. In this case, however, the necessary polarization energy is derived directly from the parallel operation of the fuel cell, and the migration of oxygen ions from the cathode to the anode/catalyst leads to a Pt-catalyst work function increase. As a result, the bond between the catalyst and the electron acceptor (in this case, CO_2_) weakens, leading to a CO production decrease. The observed electrophilic behavior of CO production agrees with previous EPOC studies [19,22,23]. In order to study the behavior of the catalytic reaction under oxygen ion removal conditions from the catalyst (anode) to the counter electrode, negative potential (U = −2 V) was applied using a potentiostat/galvanostat. The resulting decrease in the catalyst work function caused the strengthening of the Pt-CO_2_ bond and, thus, the increase in the catalytic rate both under reducing and oxidizing conditions (Figure 6).

An estimation of the activation energy of the reaction across the various conditions utilized can be accomplished via Arrhenius plots. Based on the calculated values (shown in Figure 6), it appears that the presence of oxygen ions on the catalytic surface (U = 0 V) affects the activation energy of the reaction, which takes notably increased values under both oxidizing and reducing conditions.

For the quantification of the electrochemical promotion of the CO production rate, shown in Figure 6, the rate enhancement ratio, ρ, defined by (4) [46,47], was utilized:

(4) $$\rho =\frac{r}{r_{o}}$$

where r is the electropromoted catalytic rate (U = −2 V or U = 0 V), and r_o_ is the unpromoted rate (i.e., the open-circuit catalytic rate). 

**Figure 7:**
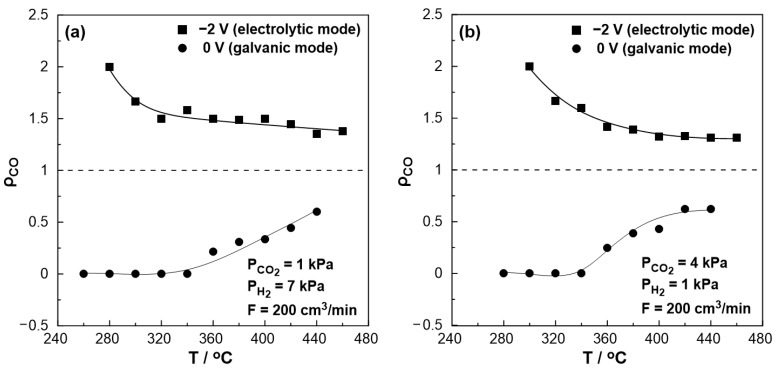
Effect of temperature under steady-state conditions on the rate enhancement ratio, ρ. (**a**) P_H_2__ = 7 kPa and P_CO_2__ = 1 kPa. (**b**) P_H_2__ = 1 kPa and P_CO_2__ = 4 kPa. F_T_ = 200 cm^3^ /min.

The effect of temperature on the rate enhancement ratio of CO under both reducing and oxidizing conditions is illustrated in Figure 7a,b, respectively. Under short circuit conditions (U = 0 V, galvanic mode) at lower temperatures (<340 °C), the observed rate enhancement ratio values are always less than unity, even approaching zero, indicating the elimination of the produced CO. On the other hand, the rate enhancement ratio for the RWGS reaction tends to be higher under negative polarization, in agreement with the electrophilic behavior of the reaction. Values up to 2 were observed at 280 °C under reducing and 300 °C under oxidizing conditions. At higher temperatures (>380 °C), the ρ value reaches a plateau with an increase of 40–50% (ρ = 1.4–1.5) under reducing and 30% (ρ = 1.3) under oxidizing conditions, even at 460 °C compared to the open circuit conditions.

**Figure 8:**
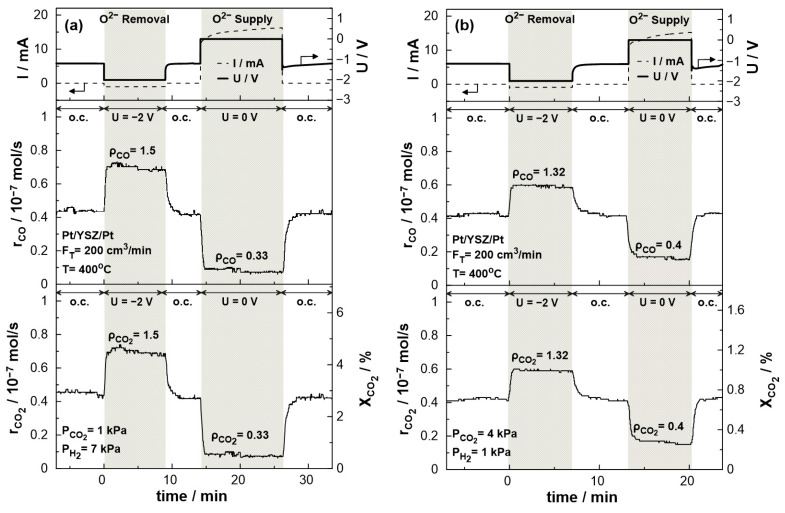
Transient effect of constant potential (U = −2 V under negative polarization, U = 0 V under short circuit conditions, and U = −1.2 V under open circuit conditions) on current (**top**), catalytic rates of CO production (**middle**) and CO_2_ consumption (**bottom** left) and CO_2_ conversion (**bottom** right). (**a**) P_H_2__ = 7 kPa and P_CO_2__ = 1 kPa. (**b**) P_H_2__ = 1 kPa and P_CO_2__ = 4 kPa. F_T_ = 200 cm^3^ /min. T = 400 °C.

Figure 8 displays the transient effect of negative polarization (U = −2 V) and short-circuit conditions (U = 0 V) on the catalytic rate of CO production and CO_2_ consumption and conversion. The experiments were carried out under both reducing (P_CO_2__:P_H_2__ = 1:7) and oxidizing (P_CO_2__:P_H_2__ = 4:1) conditions at 400 °C and a total gas flow rate of 200 cm^3^/min. The current response after the potential application is also shown in the same figure. Under electrolytic mode, i.e., negative potential (U_WR_ = −2 V), the removal of O^2-^ from the catalytic surface results in the enhancement of the production rate of CO due to the electrophilic behavior of the RWGS reaction. The CO_2_ conversion commences under open circuit conditions at a value of 2.8% with a carbon monoxide production rate of approximately 0.45 × 10^−7^ mol/s. Upon constant application of negative potential (U = −2 V), a new steady-state is achieved, with a corresponding increase in the rate of CO production. The rate enhancement ratio, ρ, for the CO production reaches the value of 1.5, denoting a significant 50% increase in the overall carbon monoxide production rate. In the promoted state, the CO_2_ conversion is approximately 4.5%. On the other hand, under galvanic mode (U = 0 V), the migration of oxygen ions to the catalytic surface leads to an enhancement of the Pt–H bond resulting in the decrease of the CO production rate to 0.1 × 10^− 7^ mol/s with a rate enhancement ratio of 0.33. Both effects of negative polarization and short-circuit conditions are fully reversible, as the rates of CO production and CO_2_ consumption return to their initial values upon interruption of the applied potential. Similar behavior was observed under oxidizing conditions. Negative polarization results in CO production increases by a factor of 1.32 (ρ_CO_ = 1.32), while under short-circuit conditions, the production rate drops by a factor of 0.4 (ρ_CO_ = 0.4) compared to the thermocatalytic production of CO at the same temperature.

**Figure 9:**
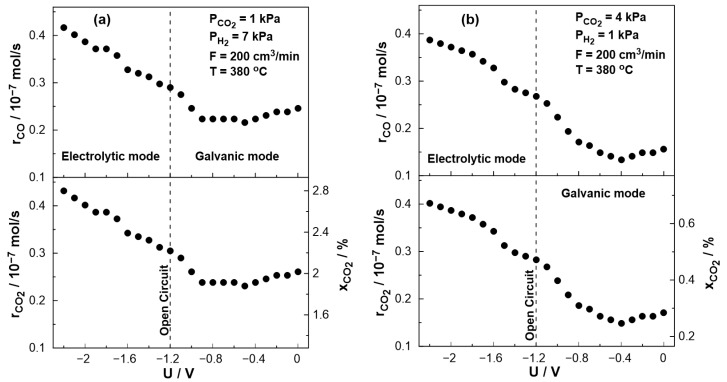
Steady-state effect of catalyst potential, U, on the catalytic rate of CO formation (**top**) and CO_2_ consumption (**bottom** left axis) and CO_2_ conversion (**bottom** right axis). (**a**) P_H_2__= 7 kPa and P_CO_2__= 1 kPa. (**b**) P_H_2__ = 1 kPa and P_CO_2__ = 4 kPa. F_T_ = 200 cm^3^/min. T= 380 °C.

Figure 9 displays the steady-state effect of catalyst potential, U, on the CO production rate, CO_2_ consumption rate, and constant temperature of T = 380 °C both under reducing and oxidizing conditions. Based on the results shown in Figure 9, the rate of CO formation increases monotonically with decreasing catalyst potential. The negative potential has a greater effect on the catalytic rate of CO (strong increase).

**Figure 10:**
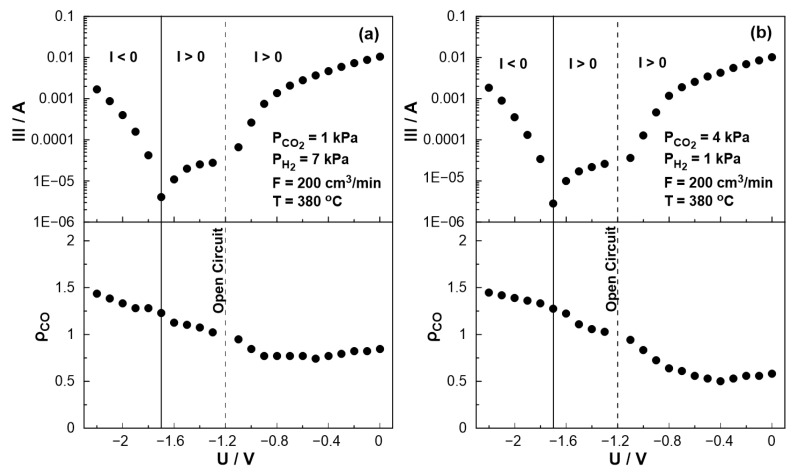
Steady-state effect of catalyst potential, U, on the rate enhancement ratio of CO formation (**bottom**) and current (Tafel plot, **top**) (**a**) P_H_2__ = 7 kPa and P_CO_2__ = 1 kPa. (**b**) P_H_2__ = 1 kPa and P_CO_2__ = 4 kPa. F_T_ = 200 cm^3^/min. T = 380 °C.

The effect of catalyst potential, U, both on the current (Tafel plot) and the rate enhancement ratio of carbon monoxide, ρ_CO_, at a constant temperature, is illustrated in Figure 10. As the potential shifts from the open circuit value (U = −1.2 V) towards zero, i.e., operating under galvanic mode (I > 0), the rate enhancement ratio of CO decreases in agreement with the observed behavior in Figure 7. As the potential approaches zero, the rate enhancement ratio of CO reaches a plateau. It is worth noting that the current, in this particular case, exhibit a positive trend that aligns with the anticipated behavior of a standard Tafel plot. On the other hand, as the catalyst potential increases towards higher absolute values in comparison to the open circuit potential (−1.7 V < U < −1.2 V), the current demonstrates a deviation from the typical Tafel plot behavior. More specifically, although one would expect to observe negative currents under these conditions, positive currents were measured for applied potentials as low as −1.7 V. This observation indicates a shift in the value of the open circuit potential, U_O.C._, which can be associated with the conducting simultaneous electrochemical and thermochemical reactions at the anodic electrode. In fact, the enhanced rate of CO production (ρ_CO_ > 1) affects both the coverage of species on the electrode surface and the fraction of the water vapor on the reactant mixture. The concentration of the produced water at the anode (both electrochemically and catalytically) could play a key role in the shift of the open circuit potential, which can also be confirmed from Nernst’s Equation for the reversible open circuit potential since all experiments were initially begun under dry reactant conditions. This is not happening in conventional SOFC studies [49,50,51,52,53], in which the anode stream usually contains a significant and constant amount of water (to ensure a specific open circuit value). It is worth noting that the above behavior was observed both under oxidizing and reducing conditions.

## 4. Conclusions

The Electrochemical Promotion of CO_2_ hydrogenation in a low-temperature SOFC-type reactor was studied using yttria-stabilized zirconia (YSZ) and a platinum (Pt) catalyst electrode. The presence of both CO_2_ and H_2_ on the anode led to a small decrease in the performance of the fuel cell (less than 15%), even at high partial pressures of CO_2_ (4 kPa). Two distinct operation modes were studied, a galvanic and an electrolytic one. In the first case, the produced power (by fuel cell operation) was used to modify the catalytic performance. The migration of oxygen ions to the catalytic surface resulted in a decrease in the CO production rate due to the enhancement of the Pt–H bond strength. Under electrolytic mode, the removal of O^2−^ from the catalytic surface resulted in the enhancement of the CO production rate (electrophilic EPOC type behavior). The rate enhancement ratio, ρ, for CO production reached a value of 1.5, denoting a 50% increase in the overall carbon monoxide production rate. The rates of CO production and CO_2_ consumption return to their initial values upon interruption of the applied potential, demonstrating the reversibility of the process. The observed electrophilic behavior of CO production agrees with previous EPOC studies on the RWGS reaction.

## Data Availability

The data that support the findings of this study are available from the corresponding author, upon reasonable request.
